# Physician professional motivation and online knowledge sharing for patient education: a perspective of motivation theory

**DOI:** 10.3389/fpubh.2025.1629272

**Published:** 2025-07-03

**Authors:** Yun Huang, Junping Guo, Yan Wen, Qihui Fan

**Affiliations:** ^1^School of Economics, Tianjin University of Finance and Economics, Tianjin, China; ^2^Department of Pharmacy, Hubei Province Corps Hospital, The Chinese Armed Police Force (CAPF), Wuhan, China; ^3^Department of General Medicine, Wuhan Third Hospital (Tongren Hospital of Wuhan University), Wuhan, China; ^4^College of Management and Economics, Tianjin University, Tianjin, China

**Keywords:** professional motivation, online knowledge sharing, online experience, offline expertise, patient education

## Abstract

**Aims:**

Drawing on motivation theory, this study aims to investigate the effect of professional motivation on online knowledge sharing for patient education with considering the contingencies of online experience and offline expertise.

**Methods:**

Based on a panel dataset comprising 11,839 physicians with 24,389 physician-month observations selected from one of leading online health platforms in China, this study conducted the fixed hierarchical regression model to test the direct and moderating effects.

**Results:**

The results show that professional motivation positively affects online knowledge-sharing quantity and quality. Meanwhile, online experience enhances the positive effects of professional motivation on the above two dimensions of online knowledge sharing. In addition, offline expertise hinders the benefits of professional motivation to online knowledge-sharing quantity.

**Conclusion:**

This study makes contributions to the literatures of motivation theory, online knowledge sharing, online and offline contexts on online health platforms, and provides implications for physicians and platform managers.

## 1 Introduction

The rapid development of digital technologies has accelerated the advancement of online healthcare services and transformed how medical knowledge is disseminated and accessed (1–3). Online health platforms (OHPs) play a pivotal role in people’s health and disease management, as physicians increasingly engage in online knowledge-sharing activities to educate patients, which is beneficial to patient outcomes and ultimately improves societal healthcare outcomes (4–6). Physicians’ knowledge sharing about OHPs is largely driven by their underlying motivations, such as their medical expertise and professional obligations (7). Therefore, motivation theory plays a critical role in understanding how different types of motivations (e.g., extrinsic and intrinsic motivation) affect physicians’ online engagement (8, 9).

Existing research has extensively explored the role of intrinsic motivation such as interest or inherent satisfaction (10, 11) and extrinsic motivation such as specific expected outcomes or external rewards (12) in online knowledge sharing. Recently, recognizing that physicians with specialized and critical medical knowledge are often driven by a sense of obligation and professional identity, even when knowledge sharing requires significant time and effort (13), scholars have shifted their attention toward professional motivation and found that they are motivated to share both free and paid health knowledge (7, 14, 15). However, this line of research has not systematically examined how professional motivation shapes the extent and quality of physicians’ knowledge-sharing behaviors. In particular, little is known about how professional motivation influences both the quantity and quality of shared content—two critical dimensions for understanding the effectiveness of physicians’ online engagement. Knowledge-sharing quantity refers to the volume of knowledge shared by physicians, while knowledge-sharing quality reflects the substantive value and utility of the shared content (16). Exploring the impact of professional motivation on knowledge-sharing quantity and quality is important in understanding the nuanced effects of professional motivation. Therefore, this study addresses this oversight by dissecting how professional motivation differentially influences these two dimensions of online knowledge sharing.

Additionally, contextual factors are increasingly recognized as critical moderators in the relationship between motivation and online knowledge sharing. According to the previous studies, online experience and offline expertise are important contingencies of physicians, which can shape their online knowledge-sharing behaviors (7, 17). Online experience refers to the accumulated knowledge and skills for using online platforms (6). Existing studies show that extensive online experience enhances physicians’ technical proficiency and confidence in online interactions (6). Conversely, offline expertise refers to the experience developed after years of clinical work and professional assessment (17). Physicians with extensive offline expertise disseminate valuable information (18) while facing considerable workloads, which limit their time and energy (19). Online experience and offline expertise tend to shape the effects of professional motivation on online knowledge sharing. However, previous studies have not simultaneously considered how physicians’ online experience and offline expertise affect the relationship between professional motivation and online knowledge sharing. Systematically considering two contingencies and guide physicians in knowledge sharing for patient education, thereby improving public health equity and welfare.

To address the above-mentioned gaps, this study aims to explore the effects of physicians’ professional motivation on the quantity and the quality of online knowledge sharing for patient education while considering the contingencies of online experience and offline expertise. Specifically, we seek to answer the following research questions:

(1) *How does physicians’ professional motivation influence online knowledge-sharing quantity and quality for patient education?* (2) *What role do online experience and offline expertise play in these relationships?*

According to motivation theory, professional motivation is the driving force in physicians sharing health knowledge on OHPs for patient education. Physicians can obtain patients’ recognition (15), enhance self-efficacy (7), pursue continuous self-growth (20), and challenge themselves (21), thereby increasing their motivation to share more knowledge online. Similarly, professional motivation, accompanied with a sense of obligation (15), confidence as authoritative guides (22), and creativity (23), also enhances physicians’ online knowledge-sharing quality. Additionally, physicians with substantial online experience demonstrate elevated perceptions of professional value due to their digital competencies and enhanced patient education capabilities (6). In this context, physicians with professional motivation are more likely to contribute a greater volume of knowledge with higher quality to support patient education. However, since distinct professional priorities are associated with varying levels of offline expertise (7, 19), the effects of professional motivation on online knowledge-sharing quantity and quality tend to be shaped by offline expertise.

The analysis in this study employed a panel dataset comprising 11,839 physicians with 24,389 physician-month observations selected from Haodf.com. The results indicate that most of the hypotheses are supported. Specifically, professional motivation is positively associated with both the quantity and quality of online knowledge sharing. Additionally, online experience further strengthens these positive effects, while offline expertise weakens the positive effect of professional motivation on online knowledge-sharing quantity. This study also makes several contributions. Firstly, this study contributes to the literature of motivation theory by focusing on professional motivation in the contexts of OHPs and identifying the formation mechanism of online knowledge sharing for patient education. Secondly, this study contributes to the literature on online knowledge sharing by revealing the positive effects of professional motivation on online knowledge-sharing quantity and quality. Thirdly, this study contributes to the literature on online and offline contexts in online knowledge sharing by uncovering the heterogeneous moderating effects of online experience and offline expertise about the impact of professional motivation on online knowledge-sharing quantity and quality.

## 2 Theory and hypotheses

### 2.1 Motivation theory

Individual needs and expectations play a crucial role in shaping behaviors (24). Drawing on this fundamental tenet, motivation defines the direction and underlying rationale of behavioral patterns, driving individuals to act in specific ways (6, 8). Motivation is commonly divided into two dimensions—extrinsic and intrinsic motivation—reflecting different attitudes and goal orientations (8). The former refers to engaging in an activity out of genuine interest or inherent satisfaction, while the latter involves undertaking an activity to achieve expected outcomes or external rewards (25).

As OHPs become increasingly prominent sources of medical knowledge (26), motivation theory has been widely applied to examine how different types of intrinsic and extrinsic motivations individually and collectively affect physicians’ online engagement. Zhuo and Wang (27) found that intrinsic (e.g., competence and autonomy satisfaction) and extrinsic motivation (e.g., economic benefits) positively influence physicians’ service behaviors. Wang et al. (9) demonstrated a crowding-out effect of informal payments on physicians’ intrinsic motivation to participate in online consultation. Beyond this classic dichotomy, recent studies have highlighted the specific role of professional motivation—a key form of intrinsic motivation—in driving the dissemination of life-critical medical knowledge by trained professionals on OHPs (14, 15). Professional motivation refers to a psychological process that influences professionals in fulfilling their goals and tasks. Zhang et al. (7) and Yang et al. (15) found that professional motivation fosters both free and paid knowledge-sharing behaviors on OHPs. However, although these studies addressed physicians’ intention, its influence on the actual performance of online knowledge-sharing behaviors remain underexplored. Unlike general users, physicians, driven by irreplaceable medical expertise and strong professional ethics, demonstrate distinct patterns of engagement on OHPs (13). To fully assess their contributions, both the quantity and quality of online knowledge sharing should be considered (28, 29), as they jointly determine the effectiveness of patient education and the sustainable development of OHPs.

This study applied motivation theory to study how physicians’ professional motivation influences both the quantity and quality of online knowledge sharing. In our framework, physicians driven by professional motivation tend to develop a stronger sense of responsibility for patient education, a desire for self-growth, and a willingness to embrace professional challenges (15), which encourages more frequent and higher-quality contributions on OHPs. Additionally, physicians’ motivating behaviors are inevitably shaped by individual difference arising from their experiences in both online and offline environments (7). Online experience strengthens the relationship between motivation and engagement by lowering technical barriers and increasing confidence in online interactions (6, 30). Offline expertise, however, may exert a dual influence: Greater clinical responsibilities lead to limit the frequency of contributions (12), whereas advanced professional seniority with deeper knowledge and practical insights can enhance the quality of shared content (19). Therefore, based on motivation theory, this study aims to explore the effects of professional motivation on online knowledge-sharing quantity and quality by considering contingencies of online experience and offline expertise.

### 2.2 Professional motivation and online knowledge sharing

Professional motivation refers to a psychological process that influences professionals (e.g., physicians and teachers) in fulfilling their goals and tasks (15). As an intrinsic motivation, professional motivation affects physicians’ online knowledge-sharing behaviors, encompassing two dimensions—online knowledge-sharing quantity and online knowledge-sharing quality (29). The former reflects the volume of knowledge shared by physicians on OHPs, while the latter pertains to the substantive value and utility of that content (16). According to motivation theory, professional motivation—characterized by heightened professional obligation, enhanced self-efficacy, and sustained persistence (7, 22)—significantly enhances physicians’ knowledge-sharing quantity and quality on OHPs.

We expect physicians’ professional motivation to be positively related to online knowledge-sharing quantity. First, professional motivation can induce physicians to use their expertise to obtain patients’ recognition, for example, votes and readings (7). The resulting fulfillment and enjoyment motivate them to make unceasing contributions to OHPs. Second, professional motivation encourages physicians to pursue continuous self-growth (20). Engaging in knowledge sharing on OHPs enables them to help patients manage health and deepen their own understanding of medical information, which, in turn, sparks their creative enthusiasm and increases the number of published articles. Third, guided by professional interests and internal goals, physicians tend to challenge themselves (21), remaining committed to frequently updating medical content and addressing patient inquiries (15), even at the cost of personal time. Based on these arguments, we propose the following hypothesis.

*H1*: Professional motivation is positively related to physicians’ online knowledge-sharing quantity.

Similarly, we expect physicians’ professional motivation to be positively related to online knowledge-sharing quality. First, professional motivation creates a strong sense of obligation in physicians to disseminate medical knowledge more extensively and efficiently through OHPs (15). This commitment ensures that their contributions meet professional standards, enhancing online knowledge-sharing quality. Second, beyond responsibility-driven factors, professional motivation boosts physicians’ confidence as authoritative guides in patient education (22), especially in the information-overloaded digital health landscape. By identifying misleading information and providing accurate clinic expertise (31), they further contribute to delivering online high-quality services. Third, professional motivation, as an intrinsically motivated orientation, is positively associated with creativity (23). Physicians with high creativity can develop innovative solutions to meet patients’ diverse healthcare needs on OHPs (7), which improves the educational value and practical utility of their efforts. Based on these arguments, we propose the following hypothesis.

*H2*: Professional motivation is positively related to physicians’ online knowledge-sharing quality.

### 2.3 The moderating effect of online experience

Online experience refers to the professionals’ accumulated knowledge and skills in using online platforms (6). Physicians with abundant online experience tend to perceive higher professional value, as they possess digital expertise and patient education skills (6). In this vein, the effects of professional motivation on online knowledge-sharing behaviors are contingent on online experience.

We expect online experience to strengthen the positive effect of professional motivation and physicians’ online knowledge-sharing quantity. With accumulated online experience, physicians not only achieve greater self-actualization by providing targeted knowledge sharing in response to high-interest health concerns (20), but they also become more skilled at developing efficient content strategies that support ongoing skill development and personal growth (32). These dual benefits jointly amplify how professional motivation drives sustained content contributions (33). In addition, physicians with extensive online experience demonstrate strong platform familiarity and advanced self-regulation skills that help optimize their knowledge-sharing practices (34, 35). Accordingly, the internal drive stemming from professional motivation is more likely to result in consistent and high-volume knowledge-sharing outputs. Thus, we propose the following hypothesis.

*H3a*: Online experience strengthens the positive relationship between professional motivation and physicians’ online knowledge-sharing quantity.

We also expect online experience to strengthen the positive effect of professional motivation and physicians’ online knowledge-sharing quality. Physicians with greater online experience are inclined to build a favorable reputation and achieve robust online socialization through consistent knowledge sharing (36). This process fosters heightened feelings of professional responsibility and self-efficacy, which supports their commitment to providing effective patient education (36, 37). Moreover, proficiency in the features and affordances of OHPs provides experienced physicians with greater flexibility in content design and delivery (6, 33). Under such circumstances, they are better positioned to match their professional values with patients’ expectations (20), further enhancing their intrinsic drive to contribute high-quality services. Thus, we propose the following hypothesis.

*H3b:* Online experience strengthens the positive relationship between professional motivation and physicians’ online knowledge-sharing quality.

### 2.4 The moderating effect of offline expertise

Offline expertise refers to the experience in delivering healthcare services and medical proficiency gained within traditional hospital settings. This expertise is often embodied in physicians’ clinical titles across four hierarchical levels—resident doctor, attending doctor, associate chief doctor, and chief doctor—signifying their professional capabilities and seniority (32). As professional priorities vary with levels of offline expertise (7, 19), offline expertise differentially moderates the relationship between physicians’ professional motivation and their online knowledge-sharing behaviors on OHPs.

We expect offline expertise to weaken the positive effect of professional motivation on physicians’ online knowledge-sharing quantity. Physicians with high levels of offline expertise (as indicated by senior clinical titles) devote greater attention to diagnosing complex medical cases to deliver specialized healthcare in offline channels (13, 38) while advancing their expertise through research and academic engagement (22). Consequently, routine health information sharing via OHPs with professional motivation is deprioritized, resulting in reduced levels of contributions. Besides, physicians in senior positions often face considerable workloads in offline hospitals, limiting the time and energy available for online engagement (19, 39), which leads to a decline in the frequency of their online knowledge sharing. Based on the above arguments, we propose the following hypothesis.

*H4a:* Offline expertise weakens the positive relationship between professional motivation and physicians’ online knowledge-sharing quantity.

Conversely, we expect offline expertise to strengthen the positive effect of professional motivation on physicians’ online knowledge-sharing quality. Motivated by a love of their work itself, accompanied with substantial professional expertise (7), physicians can cultivate a stronger sense of mission and greater confidence in their capabilities. In this context, their intrinsic professional motivation can spur the dissemination of high-quality, valuable information and experiences on OHPs (18). Additionally, by drawing on extensive face-to-face clinical experience and unique insights, physicians are better able to anticipate patients’ concerns and present in-depth medical knowledge on OHPs in a more readable manner (19). In other words, the effect of professional motivation on online knowledge-sharing quality is enhanced by offline expertise. Based on the above arguments, we propose the following hypothesis.

*H4b:* Offline expertise strengthens the positive relationship between professional motivation and physicians’ online knowledge-sharing quality.

**Figure 1:**
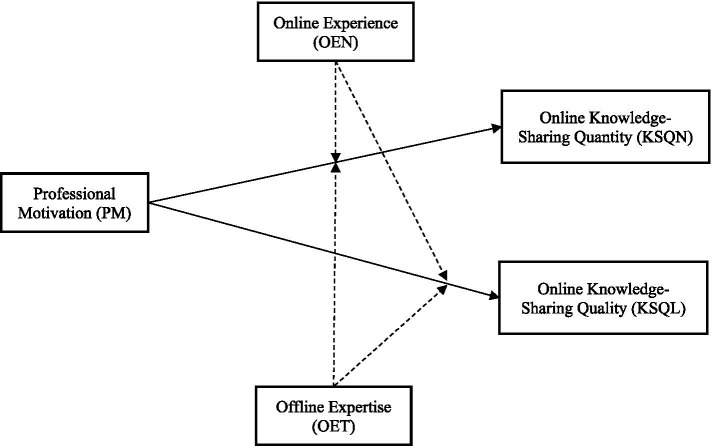
Research framework.

In summary, the research framework is presented in Figure 1.

## 3 Methods

### 3.1 Data collection

This study selected Haodf.com^1^ as our data source to its objective, real-world data that effectively mitigates self-reporting biases (40, 41). As one of leading OHPs in China, Haodf.com offers several key advantages for our research. Firstly, it hosts an extensive network of physicians and patient interactions, ensuring a robust dataset. Secondly, physicians have a dedicated article section where they can independently publish accessible health-related articles without receiving any financial compensation from the platform. These knowledge-sharing activities are systematically tracked by the platform, which records several key indicators, including the number of votes received, the number of health-related articles published, and the number of article readings. In addition, physicians’ homepages display relevant background information, such as their affiliated offline hospital, professional title, and online contribution experience, which provides valuable contextual data for this study.

To collect data, a Java-based web crawler was adopted to extract website statistics about physicians and patients over a six-month period from February to July 2017. The initial dataset included all physician profiles and their corresponding knowledge-sharing activity records available during this period. We excluded entries corresponding to physicians with incomplete profiles or without any recorded knowledge-sharing activity. This filtering process resulted in an unbalanced panel dataset comprising 11,839 physicians with 24,389 physician-month observations. This dataset enables longitudinal analysis of physicians’ online activities while accounting for variations in participation over time.

### 3.2 Measures

#### 3.2.1 Dependent variables

Online knowledge-sharing quantity refers to the volume of knowledge shared by physicians in online platforms (16), which was measured by the number of health-related articles published by physicians. Online knowledge-sharing quality reflects the substantive value and utility of the shared content in online platforms (16). It was measured by the number of article readings.

#### 3.2.2 Independent variables and moderators

Professional motivation reflects a psychological process that influences professionals in fulfilling their goals and tasks (7, 15). The number of votes received by physicians was adopted to measure professional motivation. As physicians do not receive financial incentives for publishing online articles, voting reflects patients’ recognition of their voluntary knowledge-sharing efforts (7). In this context, such behaviors are primarily driven by professional motivation, including a sense of obligation to contribute to patient education and a desire for self-development. Online experience reflects the professionals’ accumulated knowledge and skills in using online platforms (6), which was measured by the opening time of physicians. Offline expertise refers to the experience in delivering healthcare services and medical proficiency gained within traditional hospital settings (17). It was measured by the offline titles of physicians. According to the hospital title hierarchy in China, we included three dummy variables for four levels, ordered from lowest to highest: resident physician, attending physician, associate chief physician, and chief physician (17).

#### 3.2.3 Control variables

In studies on physician online knowledge sharing and patient education, previous scholars suggest that gifts and likes may be seen as factors influencing the online knowledge-sharing behaviors (17, 42); therefore, we choose them as control variables. Gifts were measured by the number of online gifts from patients. Likes were measured by the number of online loves from patients.

To reduce skewness, we used the logarithm of all variables except offline expertise. Table 1 presents an overview of all variables in this study.

**Table 1:** The overview of all variables.

Variables	Measurements	Mean	SD	Min	Max
Online knowledge-sharing quantity	The number of health-related articles published by physicians	2.011	1.280	0.000	7.440
Online knowledge-sharing quality	The number of article readings	7.899	1.108	2.079	12.753
Professional motivation	The number of votes physicians received	2.634	1.239	0.000	6.911
Online experience	The opening time of physicians	7.270	0.798	3.332	8.010
Offline expertise	The offline titles of physicians	3.098	0.869	1.000	4.000
Gifts	The number of online gifts from patients	2.506	1.385	0.000	7.920
Likes	The number of online likes from patients	3.789	2.030	0.000	10.140

### 3.3 Analytic strategy

Considering the inefficiency and estimated bias of the ordinary least squares regression model, this study conducted the fixed hierarchical regression model to test the direct and moderating effects (43, 44). To test our hypotheses, we introduced Equations 1, 2 to estimate the effects of professional motivation (PM) on online knowledge-sharing quantity (KSQN) and online knowledge-sharing quality (KSQL) with the contingencies of online experience (OEN) and offline expertise (OET).


(1) $$\begin{array}{l} \mathrm{KSQ}N_{\mathrm{it}}=\alpha_{0}+\alpha_{1}\mathrm{Gif}t_{\mathrm{it}}+\alpha_{2}\mathrm{Lik}e_{\mathrm{it}}+\alpha_{3}PM_{\mathrm{it}}+\alpha_{4}\mathrm{OE}N_{\mathrm{it}}\\ +\alpha_{5}\mathrm{OE}T_{\mathrm{it}}+\alpha_{6}PM_{\mathrm{it}}\times \mathrm{OE}N_{\mathrm{it}}+\alpha_{7}PM_{\mathrm{it}}\times \mathrm{OE}T_{\mathrm{it}}+\mu_{\mathrm{it}}\; \end{array}$$

(2) $$\begin{array}{l} \mathrm{KSQ}L_{\mathrm{it}}=\beta_{0}+\beta_{1}\mathrm{Gif}t_{\mathrm{it}}+\beta_{2}\mathrm{Lik}e_{\mathrm{it}}+\beta_{3}PM_{\mathrm{it}}+\beta_{4}\mathrm{OE}N_{\mathrm{it}}\\ +\beta_{5}\mathrm{OE}T_{\mathrm{it}}+\beta_{6}PM_{\mathrm{it}}\times \mathrm{OE}N_{\mathrm{it}}+\beta_{7}PM_{\mathrm{it}}\times \mathrm{OE}T_{\mathrm{it}}+\nu_{\mathrm{it}} \end{array}$$

where i indicates the number of observations, the α and β parameters are the coefficients that can be estimated in the hierarchical regression model, and the μ and ν parameters are the error terms in each equation.

## 4 Results

### 4.1 Regression analysis

Our hypotheses were tested using hierarchical regression, which is widely applied to test moderating effects (45). The results are presented in Table 2.

**Table 2:** Results of hierarchical regression.

**Online knowledge-sharing quantity (KSQN)**	**Model 1**	**Model 2**	**Model 3**	**Model 4**
Professional motivation (PM)	0.084^***^ (0.008)	-0.472^***^ (0.062)	0.081^***^ (0.025)	-0.497^***^ (0.062)
Online experience (OEN)		0.150^***^ (0.021)		0.084^***^ (0.023)
PM×OEN		0.067^***^ (0.008)		0.083^***^ (0.009)
Offline expertise (OET)			0.198^***^ (0.020)	0.178^***^ (0.022)
PM×OET			-0.017^*^ (0.007)	-0.039^***^ (0.008)
Gifts	0.387^***^ (0.012)		0.370^***^ (0.012)	0.284^***^ (0.013)
Likes	-0.126^***^ (0.008)		-0.101^***^ (0.008)	-0.034^***^ (0.009)
Constant	1.298^***^ (0.019)		0.786^***^ (0.061)	0.294 (0.152)
R^2^	0.100	0.129	0.109	0.132
**Online knowledge-sharing quality (KSQL)**	**Model 5**	**Model 6**	**Model 7**	**Model 8**
Professional motivation (PM)	0.151^***^ (0.007)	-0.168^**^ (0.055)	0.116^***^ (0.022)	-0.163^**^ (0.055)
Online experience (OEN)		0.161^***^ (0.019)		0.171^***^ (0.020)
PM×OEN		0.036^***^ (0.007)		0.036^***^ (0.008)
Offline expertise (OET)			0.020 (0.018)	-0.034 (0.019)
PM×OET			0.007 (0.007)	0.001 (0.007)
Gifts	0.284^***^ (0.011)	0.200^***^ (0.011)	0.279^***^ (0.012)	0.200^***^ (0.011)
Likes	-0.162^***^ (0.007)	-0.091^***^ (0.007)	-0.101^***^ (0.011)	-0.093^***^ (0.007)
Constant	7.404^***^ (0.017)	6.317^*^ (0.134)	7.361^***^ (0.054)	6.331^***^ (0.134)
R^2^	0.075	0.099	0.075	0.099

*N* = 11,839; ^*^p < 0.050, ^**^p < 0.010, ^***^p < 0.001; standard errors are in parentheses.

**Figure 2:**
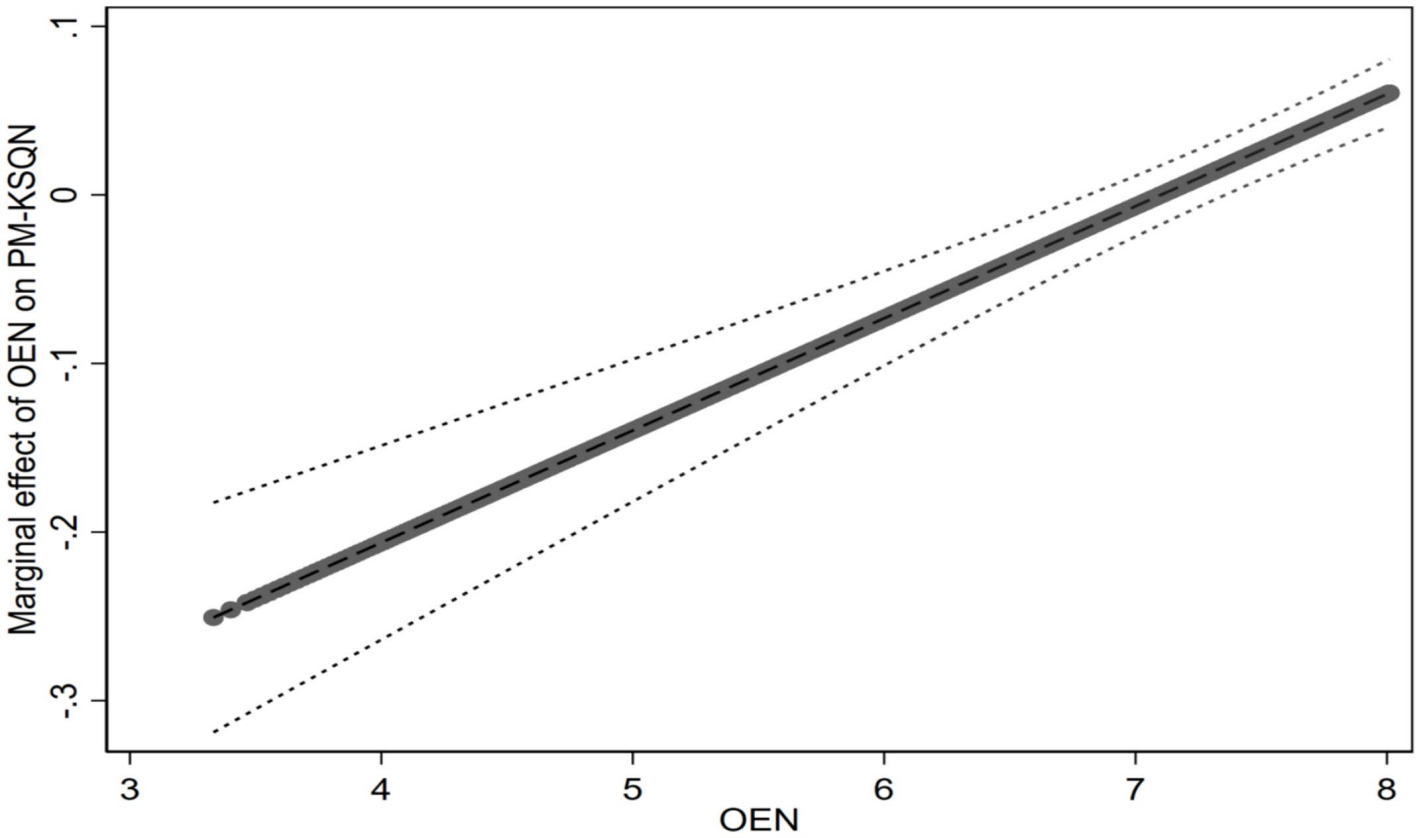
The moderating effect of online experience (OEN) on the relationship between professional motivation (PM) and online knowledge-sharing quantity (KSQN).

**Figure 3:**
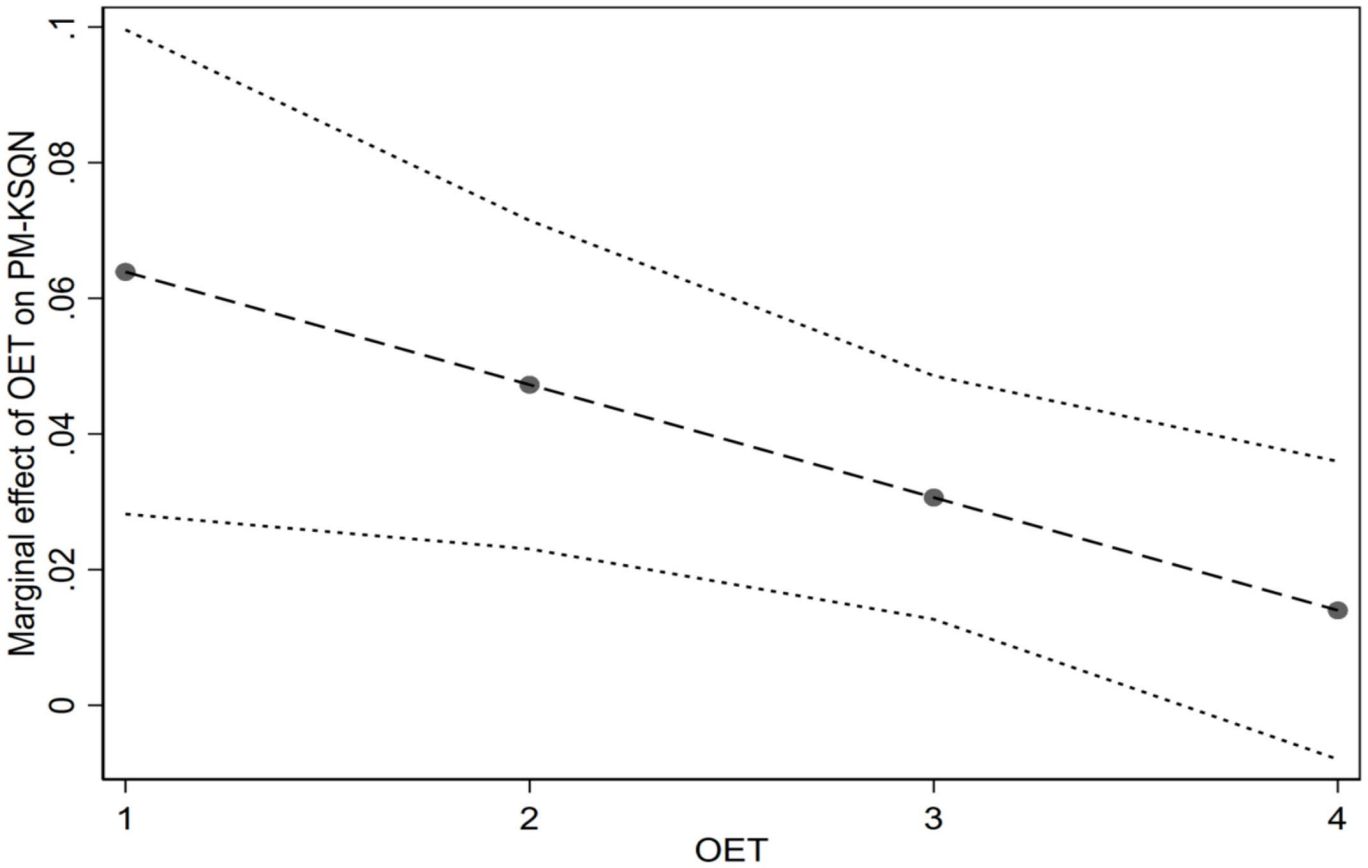
The moderating effect of offline expertise (OET) on the relationship between professional motivation (PM) and online knowledge-sharing quantity (KSQN).

Model 1 shows that professional motivation is positively and significantly related to online knowledge-sharing quantity (*β* = 0.084, *p* < 0.001). Thus, H1 is supported. The coefficient of interaction term (PM × OEN) in Model 2 is positive and significant (*β* = 0.067, *p* < 0.001). Following the suggestions of Meyer et al. (46), we plotted the marginal effect of professional motivation on online knowledge-sharing quantity at different levels of online experience in Figure 2. As the value of online experience increases from low to high, the effect of professional motivation on knowledge-sharing quantity becomes stronger. Therefore, H3a is supported. Model 3 shows that interaction term (PM × OET) is negatively and significantly associated with online knowledge-sharing quantity (*β* = −0.017, *p* < 0.050). Figure 3 illustrates that the effect of professional motivation on online knowledge-sharing quantity becomes weaker with the value of offline expertise increasing from low to high. Therefore, H4a is supported. Following the suggestions of Guo et al. (47), our study applied full models to further test our hypotheses testing in Model 4, and the results are consistent with Model 2 and Model 3.

**Figure 4:**
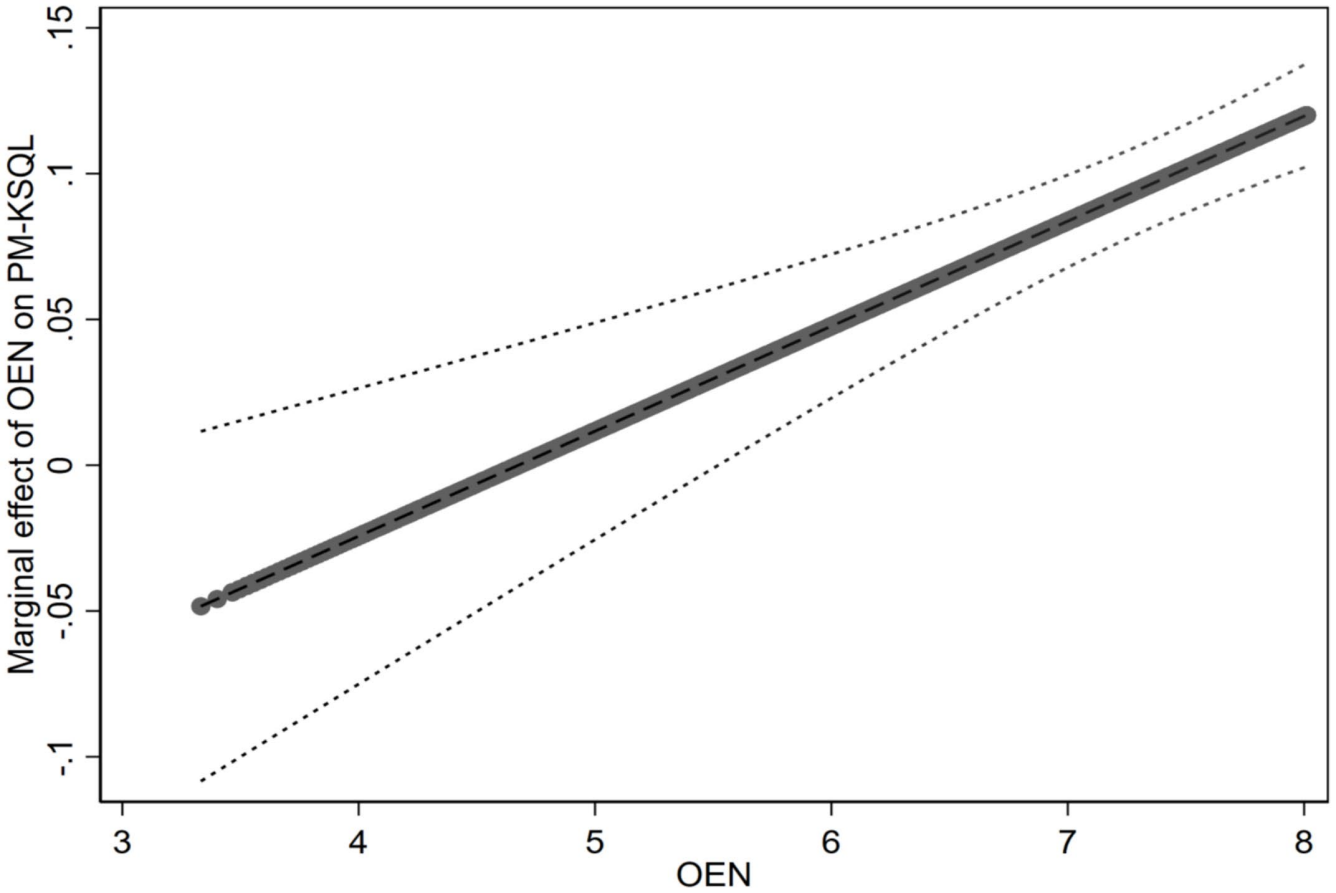
The moderating effect of online experience (OEN) on the relationship between professional motivation (PM) and online knowledge-sharing quality (KSQL).

Model 5 shows that professional motivation is positively and significantly related to online knowledge-sharing quality (*β* = 0.151, *p* < 0.001), thereby supporting H2. In Model 6, the interaction term (PM × OEN) is positively and significantly related to online knowledge-sharing quality (*β* = 0.036, p < 0.001). We also plotted the figure about the moderating effect of online experience. Figure 4 indicates that the positive relationship between professional motivation and online knowledge-sharing quality becomes stronger with an increase in online experience. Thus, H3b is supported. Model 7 shows that the coefficient of interaction term (PM × OET) on online knowledge-sharing quality is not significant (*β* = 0.007, *p* > 0.050). Thus, H4b is not supported. Full models were applied to test our hypotheses in Model 8, and the results are consistent with Model 6 and Model 7.

### 4.2 Supplementary analyses

To test the robustness of our regression results, according to whether online experience (OEN) and offline expertise (OET) are above or below the mean value, we divided the sample into subsamples (OEN_high_ vs. OEN_low_, OET_high_ vs. OET_low_) to conduct regression between the independent variable and dependent variable following the suggestion of previous studies (48, 49). For moderating effect of online experience, the effects of professional motivation on online knowledge-sharing quantity (*β*_high_ = 0.228, *p* < 0.001 vs. *β*_low_ = 0.066, *p* < 0.001) and online knowledge-sharing quality (*β*_high_ = 0.158, p < 0.001 vs. *β*_low_ = 0.116, *p* < 0.001) are stronger in high levels of online experience. For the moderating effect of offline expertise, the slope is flatter under higher levels of offline expertise in the relationship between professional motivation on online knowledge-sharing quantity (*β*_high_ = −0.041, *p* < 0.010 vs. *β*_low_ = 0.107, *p* < 0.001). All these results are consistent with the regression analysis, which further supports the interaction hypotheses.

To further test the robustness of our results, random effects regression models were conducted following previous studies (50, 51). The results are presented in Table 3. The effect of professional motivation on online knowledge-sharing quantity is positive and significant (*β* = 0.082, *p* < 0.001) in Model 9, which supports H1. Model 13 indicates that professional motivation is positively and significantly related to online knowledge-sharing quality (*β* = 0.151, *p* < 0.001), thereby supporting H2. Model 10 and Model 14 verified the moderating roles of online experience about the effect professional motivation on online knowledge-sharing quantity (*β* = 0.068, *p* < 0.001) and online knowledge-sharing quality (*β* = 0.036, *p* < 0.001). Thus, H3a and H3b are supported. The interaction term (PM × OET) in Model 11 is significantly related to online knowledge-sharing quantity (*β* = −0.016, *p* < 0.050), while the interaction term (PM × OET) in Model 15 is insignificantly related to online knowledge-sharing quality (*β* = 0.007, *p* > 0.050). Therefore, H4a is supported but H4b is rejected. The full models in Model 12 and Model 16 align with the results from the respective separate models. In summary, the results are similar to fixed effects, and our results are robust.

**Table 3:** Results of regression models with random effects.

**Online knowledge-sharing quantity (KSQN)**	**Model 9**	**Model 10**	**Model 11**	**Model 12**
Professional motivation (PM)	0.082^***^ (0.008)	-0.486^***^ (0.062)	0.075^***^ (0.025)	-0.510^***^ (0.062)
Online experience (OEN)		0.151^***^ (0.021)		0.084^***^ (0.023)
PM×OEN		0.068^***^ (0.008)		0.084^***^ (0.009)
Offline expertise (OET)			0.198^***^ (0.020)	0.178^***^ (0.022)
PM×OET			-0.016^*^ (0.007)	-0.038^***^ (0.008)
Gifts	0.394^***^ (0.012)	0.285^***^ (0.013)	0.377^***^ (0.012)	0.288^***^ (0.013)
Likes	-0.136^***^ (0.008)	-0.044^***^ (0.008)	-0.110^***^ (0.008)	-0.041^***^ (0.009)
Constant	1.320^***^ (0.019)	0.326^*^ (0.153)	0.806^***^ (0.061)	0.309^*^ (0.153)
R^2^	0.097	0.127	0.107	0.131
**Online knowledge-sharing quality (KSQL)**	**Model 13**	**Model 14**	**Model 15**	**Model 16**
Professional motivation (PM)	0.151^***^ (0.007)	-0.170^**^ (0.055)	0.115^***^ (0.022)	-0.165^**^ (0.055)
Online experience (OEN)		0.161^***^ (0.019)		0.172^***^ (0.020)
PM×OEN		0.036^***^ (0.007)		0.036^***^ (0.008)
Offline expertise (OET)			0.021 (0.018)	-0.034 (0.019)
PM×OET			0.007 (0.007)	0.002 (0.007)
Gifts	0.286^***^ (0.011)	0.200^***^ (0.011)	0.280^***^ (0.011)	0.200^***^ (0.011)
Likes	-0.164^***^ (0.007)	-0.092^***^ (0.007)	-0.157^***^ (0.007)	-0.094^***^ (0.008)
Constant	7.406^***^ (0.017)	6.314^*^ (0.134)	7.363^***^ (0.054)	6.329^***^ (0.134)
R^2^	0.075	0.099	0.076	0.100

*N* = 11,839; ^*^p < 0.050, ^**^p < 0.010, ^***^p < 0.001; standard errors are in parentheses.

## 5 Discussion

Drawing on motivation theory, this study examines the relationship between physicians’ professional motivation for patient education and their online knowledge-sharing behaviors on OHPs. Using a six-moth panel dataset of 11,839 physicians, the findings confirm that professional motivation significantly enhances both the quantity and quality of online knowledge sharing, with online experience further strengthening these positive effects. Interestingly, while offline expertise weakens the positive effect of professional motivation on online knowledge-sharing quantity, this study finds no significant empirical support for its moderating role in online knowledge-sharing quality. One possible explanation lies in the hierarchical structure of Chinese hospitals, where physicians with extensive offline expertise often take on additional responsibilities, such as administrative duties and teaching tasks (19, 52), which divert their time and attention from refining shared knowledge. Consequently, even with high professional motivation, their online knowledge-sharing quality may not be shaped by offline expertise. In addition, using the number of article readings as a proxy for knowledge-sharing quality could introduce bias, as highly specialized content contributed by senior physicians, although often of high quality, may naturally attract fewer patients compared to common health topics (53). This limitation is likely to obscure the potential moderating effect of offline expertise. In the following sections, we will discuss the theoretical and practical implications, limitations, and future directions for research.

### 5.1 Theoretical implications

This study makes several theoretical contributions. First, it contributes to the literature on motivation theory by focusing on professional motivation in patient education and identifying the formation mechanism of online knowledge sharing. Previous studies have shown that physicians driven by professional motivation are more willing to engage in knowledge sharing on OHPs (7, 15). Our study confirms the significant intrinsic impact of professional motivation on physicians’ online knowledge-sharing behaviors, supporting the existing literature on its role in patient education (13, 14). Moreover, our study explores how external factors, including both online experience and offline expertise, shape physicians’ internal motivational processes. Therefore, this study develops a comprehensive framework from the lens of motivation theory to better understand the formation mechanism of online knowledge sharing for patient education.

Second, this study enriches the literature of online knowledge sharing by revealing the positive effects of professional motivation on online knowledge-sharing quantity and quality. Although the extant research has widely explored their engagement with OHPs, it has primarily focused on the types of content shared (e.g., free vs. paid) or behavioral intentions (15, 27, 54), with relatively little attention given to the different dimensions of online knowledge sharing. In the context of OHPs, where patients rely on contributed information for making health-related decisions (55), understanding the scope and value of the knowledge being shared is particularly important (56). In this vein, our study quantifies online knowledge sharing in terms of quantity and quality, as well as examining how professional motivation influences each dimension. The results show that professional motivation significantly enhances both dimensions, although the strength of its effect varies across individuals (7). By incorporating the dual dimensions of quantity and quality into online knowledge sharing, this study provides a nuanced and outcome-oriented perspective on physician engagement in online knowledge sharing.

Third, this study underscores the importance of online and offline contexts in online knowledge sharing by uncovering the moderating effects of online experience and offline expertise. While OHPs serve as a complementary channel to offline healthcare, physicians often act as knowledge providers across both contexts (57). In this vein, their professional experiences in one domain may influence their engagement in the other, generating cross-contextual spillover effects (56). However, recognizing the limitations of isolated analyses of online and offline factors in prior research, we complement the literature by introducing online experience and offline expertise into a unified framework as co-existing contextual contingencies. Our findings show that online experience strengthens the effects of professional motivation on both the quantity and quality of online knowledge sharing, whereas offline expertise dampens its impact on quantity. In doing so, this study expands the boundary conditions of motivation theory by shedding light on the dynamic interplay between physicians’ digital participation and their professional expertise.

### 5.2 Practical implications

This study also provides valuable implications for medical practitioners and platform managers. Given that professional motivation is positively related to both the quantity and quality of online knowledge sharing, physicians need to cultivate a strong professional identity. Specifically, they should recognize that online patient education—explaining medical concepts, addressing common misconceptions, and providing evidence-based health advice—is an integral part of their professional responsibilities rather than an additional burden (17). This shift can inspire greater engagement in online knowledge-sharing efforts. Also, physicians can boost their motivation by engaging with patient feedback online through responding to comments and participating in Q&A forums. Such interaction can help tailor content to address real concerns and reinforces their commitment to online knowledge sharing.

In addition, platforms are supposed to shoulder responsibilities for supporting physicians’ adaptation to digital engagement (58), particularly given the heterogeneity among physician groups. On the one hand, platform designers should implement targeted strategies to improve physicians’ proficiency in online interactions in light of the positive moderating role of online experience. For example, by analyzing multi-dimensional patient feedback, these designers can leverage AI-driven algorithms to identify effective knowledge-sharing practices. By doing so, physicians can gain deeper insights into patient preferences, enabling them to publish high-quality articles efficiently with minimal time investment. Moreover, platform-based initiatives, such as mentorship programs that connect experienced online contributors with novices, and training modules designed to enhance digital communication skills for patient education, can further strengthen physicians’ proficiency in online interactions and support sustained knowledge-sharing behaviors. On the other hand, platforms need to recognize and counterforce against the negative role of offline expertise on knowledge sharing. Teamwork serves as a viable solution to this challenge by, for example, allowing physicians to establish verified virtual team-based accounts (40). Within this framework, senior physicians with high clinic titles provide authoritative mentorship, sharing professional insights and practical experience, while the other junior physicians contribute by assisting with content creation and digital engagement. This division of labor creates a synergistic relationship, ultimately maintaining professional standards and online knowledge-sharing continuity.

### 5.3 Limitations and research directions

There are several limitations and open questions that are worthwhile to further research. First, the exclusive reliance on data from Haodf.com in China raises questions regarding the generalizability of our findings. Given the professional nature of physicians, the main finding that professional motivation positively influences online knowledge-sharing behaviors is likely to be broadly applicable across various OHPs. However, effects such as the moderating effect of offline expertise may be shaped by differences in healthcare system structures and professional hierarchies internationally. These potential variations call for future research to build upon this study by incorporating multiple OHPs across diverse digital ecosystems. Second, while our study mitigated endogeneity by employing several supplementary analyses, potential endogeneity concerns cannot be entirely eliminated. To strengthen causal identification, future studies could adopt methods such as difference-in-differences (DID), quasi-experimental designs, and synthetic control methods (59). Third, we primarily studied the contingent role from the perspective of physicians’ attributes, particularly online experience and offline expertise. However, exploring alternative moderators may provide deeper insights into physicians’ knowledge-sharing dynamics, for example, gamification settings (60) and income (15). They are other avenues for future studies. Finally, using article readings as a proxy for knowledge-sharing quality may underestimate the value of highly specialized content that naturally attracts a narrower patient audience, leading to potential measurement bias. Similarly, measuring online experience by physicians’ opening time may fail to capture the intensity and nature of online engagement, such as the frequency, recency, and diversity of online activities. Future research could incorporate multi-dimensional metrics or conduct experimental research designs to provide more nuanced assessments of these constructs.

## Data Availability

The datasets presented in this article are not readily available because the data is available for request. Requests to access the datasets should be directed to fanqihui_1019@tju.edu.cn.

## Footnotes

^1^ www.haodf.com
